# An Integrated Korean Biodiversity and Genetic Information Retrieval System

**DOI:** 10.1186/1471-2105-9-S12-S24

**Published:** 2008-12-12

**Authors:** Jeongheui Lim, Jong Bhak, Hee-Mock Oh, Chang-Bae Kim, Yong-Ha Park, Woon Kee Paek

**Affiliations:** 1 Korean BioInformation Center, Korea Research Institute of Bioscience and Biotechnology, Daejeon, 305-806, Korea; 2 Biological Resource Center, Korea Research Institute of Bioscience and Biotechnology, Daejeon, 305-806, Korea; 3 Major in Life Science, Sangmyung University, Seoul, 110-743, Korea; 4 Department of Applied Microbiology, Yeungnam University, Kyeongsan, 712-749, Korea; 5 Division of Natural History, National Science Museum, Daejeon, 305-705, Korea

## Abstract

**Background:**

On-line biodiversity information databases are growing quickly and being integrated into general bioinformatics systems due to the advances of fast gene sequencing technologies and the Internet. These can reduce the cost and effort of performing biodiversity surveys and genetic searches, which allows scientists to spend more time researching and less time collecting and maintaining data. This will cause an increased rate of knowledge build-up and improve conservations. The biodiversity databases in Korea have been scattered among several institutes and local natural history museums with incompatible data types. Therefore, a comprehensive database and a nation wide web portal for biodiversity information is necessary in order to integrate diverse information resources, including molecular and genomic databases.

**Results:**

The Korean Natural History Research Information System (NARIS) was built and serviced as the central biodiversity information system to collect and integrate the biodiversity data of various institutes and natural history museums in Korea. This database aims to be an integrated resource that contains additional biological information, such as genome sequences and molecular level diversity. Currently, twelve institutes and museums in Korea are integrated by the DiGIR (Distributed Generic Information Retrieval) protocol, with Darwin Core2.0 format as its metadata standard for data exchange. Data quality control and statistical analysis functions have been implemented. In particular, integrating molecular and genetic information from the National Center for Biotechnology Information (NCBI) databases with NARIS was recently accomplished. NARIS can also be extended to accommodate other institutes abroad, and the whole system can be exported to establish local biodiversity management servers.

**Conclusion:**

A Korean data portal, NARIS, has been developed to efficiently manage and utilize biodiversity data, which includes genetic resources. NARIS aims to be integral in maximizing bio-resource utilization for conservation, management, research, education, industrial applications, and integration with other bioinformation data resources. It can be found at .

## Background

Plant and animal specimen data with surveys and observational data stored in museums and herbaria provide a vast information resource; these data include not only the historic information going back several hundred years, but also present day information on the locations of these entities [1]. The availability of this specimen data in on-line databases is greatly improving science and reducing cost and effort by providing for more efficient and effective biological surveys, which allow scientists to spend more time on research [2].

Traditionally, collections in museums and herbaria were made with only one main purpose in mind, taxonomic study, but their long-term mission has been to document biodiversity and its distribution through time and space for research, education, and service to the public [3]. The introduction of computer databases has opened up this vast data storage to many new uses [4]. These uses include biogeographic studies [5], conservation planning [6], reserve selection [7], climate change studies [8,9], species translocation studies [10], etc.

The Internet's development has allowed new opportunities for interchanging data. Until the Species Analyst project [11], which began in the late 90's, there had been few successful electronic data interchange projects that utilized the Internet. Since then, a number of distributed projects have been initiated: the Mammal Networked Information System (MaNIS) [12], the World Network on Biodiversity (CONABIO) [13], the Australian Virtual Herbarium (AVH) [14], and the European Natural History Specimen Information Network (ENHSIN) [15]. Furthermore, a number of international cooperative projects (e.g., GBIF data portal, Species 2000, Consortium for the Barcode of Life (CBOL), Encyclopedia of Life (EOL), Ocean Biogeographic Information System (OBIS), Scratchpads [16], and WikiSpecies [17]) have recently begun to link and distribute biodiversity data among countries and international organizations. A general trend in bioscience is that advanced bioinformatics analysis and link-up tools are overcoming the challenges of different database types [18,19]. For example, NCBI's databases contain information from molecular to species in different layers [20]. These layers are now becoming linked and integrated through genetic information from fast sequencing projects such as metagenomics; this process also accelerates biotechnological applications. The most important aspect of integration is that once these systems are deployed world wide, it will be possible for machines to process biodiversity information automatically, assuming the data are accurate. This will also accelerate standardization and raise the efficiency of the process of applying biodiversity studies by many magnitudes.

The biodiversity databases in Korea have been scattered among several institutes and local natural history museums, and the data were made up of heterogeneous types of databases with different formats and properties; a centralized standardized portal that enabled access to biodiversity information was necessary. A web portal will be able to speed up the investigation of complicated biological inquiries, allow researchers to develop new knowledge by analyzing large data sets, and provide the appropriate analytical tools [21]. For example, identification studies of predominant areas for conservation [22] and impacts of climate change across natural systems [23] will be increased through the accessibility of large, integrated databases.

We built NARIS, a database and national website for biodiversity, to manage and utilize the biodiversity resources in Korea and beyond. This system can be used in other nations with minimum modifications and language translations. At its core, NARIS is a biodiversity database, but it includes links to molecular and genetic information from the NCBI databases. NARIS aims to promote conservation, management, education, research, and industrial applications in biodiversity. In particular, with the advancements in fast genome sequencing technology, we expect that most of the common species will be completely sequenced before 2015. An integrated biodiversity management system such as NARIS will provide a platform that directly integrates the vast amount of genetic diversity information in the existing species diversity information.

## Methods

### System architecture

**Figure 1:**
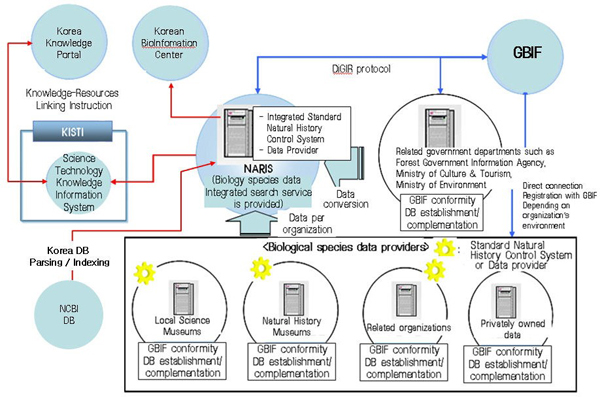
**NARIS System Architecture**. The data from several institutes and museums in Korea was integrated with NARIS by DiGIR protocol for data sharing between data providers, portal engine, GBIF, Korean BioInformation Center, Korea Science Technology Knowledge Information System, and Korea Knowledge Portal.

NARIS was built through the cooperation of many organizations from industry, universities, and research institutes in Korea. The participants are presented in Table 1. As shown in Figure 1, the biodiversity data stored in each organization was integrated with the NARIS database using the Darwin Core2.0 metadata standard along with DiGIR protocol [24], which is a request and response message format for communication between data provider, portal engine, and applications. By linking the metadata and the existing databases already available in each organization, any addition, deletion, or modification of the databases is automatic, so the metadata are integrated into NARIS. The adoption of the DiGIR protocol enables data sharing between GBIF, Korean BioInformation Center (KOBIC), Korea Science Technology Knowledge Information System (KISTI), and Korea Knowledge Portal.

**Table 1:** Participants of NARIS. As of August 2008, the data generated from 12 institutes and natural history museums in Korea are standardized with the Darwin Core2.0 format and integrated with NARIS to share and exchange the data.

Organizations	Records of Data
National Science Museum	137,680

Korea National Arboretum	980,000

Gyeryongsan Natural History Museum	4,000

Kyunghee University Natural History Museum	6,181

Mokpo Natural History Museum	17,754

Hannam University Natural History Museum	5,000

Seodaemun Museum of Natural History	2,010

Ewha Womans University Natural History Museum	5,050

Jeonam Maritime & Fisheries Science Museum	2,000

Folklore & Natural History Museum Jeju Special Self-Governing Province	54,588

Chungnam University Natural History Museum	1,005

Busan Marine Natural History Museum	2,275

**Figure 2:**
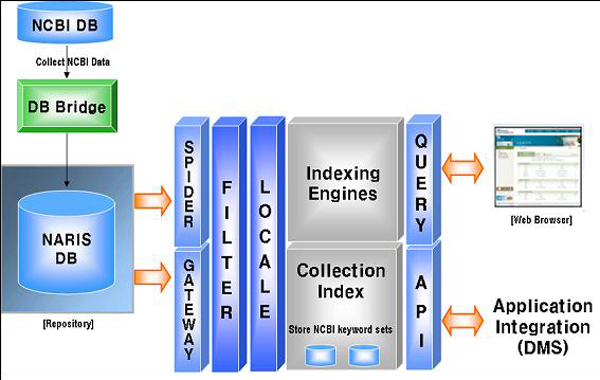
NCBI Data Search and Link Engine.

Figure 2 shows the steps for linking NARIS keywords to NCBI's genetic and taxonomic data. First, NARIS retrieves and stores relevant NCBI data in a table using WebCrawler and parsing tool. Second, NCBI's search fields are selected from the NARIS database via SPIDER and Gateway; the search fields are type, category, scientific name, affiliation, sequence, and abstract. Third, the Filter converts the binary data into text. Fourth, the keyword sets are extracted at the Locale stage and stored by Collection Index schema. When a user inputs a search word in the web browser, this is converted to the database Query, and a search engine finally shows the results from the Collection Index.

The NARIS system uses an XML-based database connection module. The search engine supports a logical calculator, wildcards, Processor, API (C, Java-based), web-based management, and database connection with WAS and Oracle. It is equipped with several functions: a multi-database search, a document filter, a synonym/thesaurus dictionary, morpheme analyses, popular and recommended keywords, and statistical analyses. The J2EE (Java 2 Platform, Enterprise Edition) technology has been used for web service, as well as JDBC (Java Database Connectivity), using a database connection pool.

### Data format and protocol

**Figure 3:**
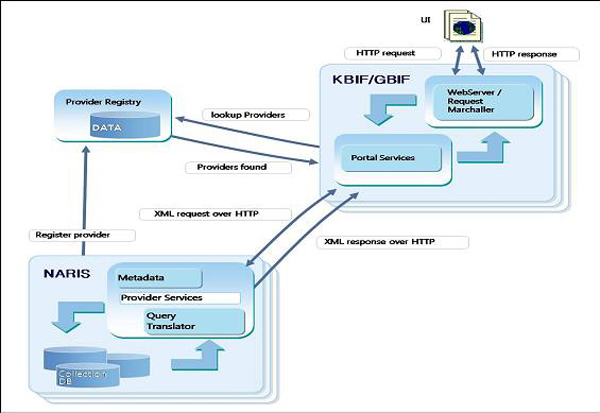
**Schematic Diagram of the DiGIR Protocol**. The DiGIR protocol is installed in each data provider's server, and the data are extracted after matching with provider databases. The data are saved in a temporary storage of Provider Registry by XML, and then they are transmitted to each portal system, along with corresponding metadata collected through the DiGIR software installed in each data provider's web server.

The NARIS data format includes 79 fields, with additional field extensions in addition to the Darwin Core2.0 standard format, to incorporate required variables for the comprehensive interchange of biodiversity data. Figure 3 shows the schematic diagram of the DiGIR protocol, which allows NARIS data to be integrated with other informatics initiatives, such as GBIF and OBIS, to retrieve, link, and integrate the metadata from each relevant organization. The DiGIR protocol allows more precise data searches because it is designed to reduce information overload and extract common characteristics from all data sources that are shown in different formats.

## Results and discussion

### User interface

**Figure 4:**
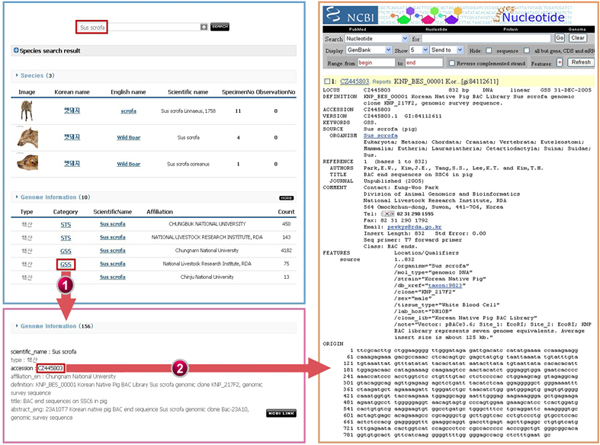
**Sample of Linked Genetic Information for *Sus scrofa *(pig)**. The integrated information for *Sus scrofa*, which included taxonomic data and genetic data from NCBI databases, can be accessed in a single search result page.

The NARIS user-interface mainly consists of a search page and an integrated search result page. The search page has a single input window, and the search result page shows integrated information with various items: species descriptions, the introduction of protective species and endangered species, natural heritages, molecular and genetic information, geographical distribution, specimen and observational data, biodiversity related organizations, videos, images, news, references, etc. As shown in Figure 4, by the integration with NCBI's genetic data, NARIS allows users to search genetic information (e.g., nucleotide, protein, sequence) in a single search result page.

### Unique functionalities

**Figure 5:**
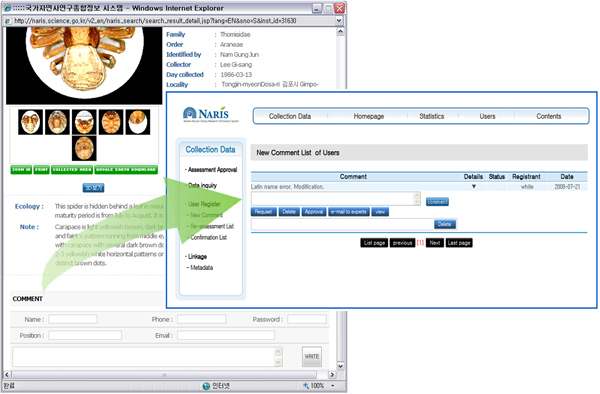
**Data Error Report Function**. A user can report a datum error in the comment box on NARIS's web site. Then the message is reported to a NARIS administrator.

**Figure 6:**
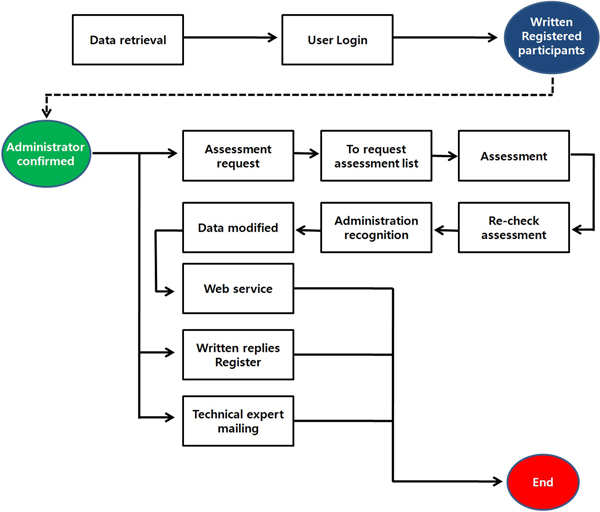
**Data Error Management Procedure**. An incorrect datum reported from a user on NARIS's web site is reported to the NARIS administrator; the subject area experts assess it; and the incorrect datum is revised.

**Figure 7:**
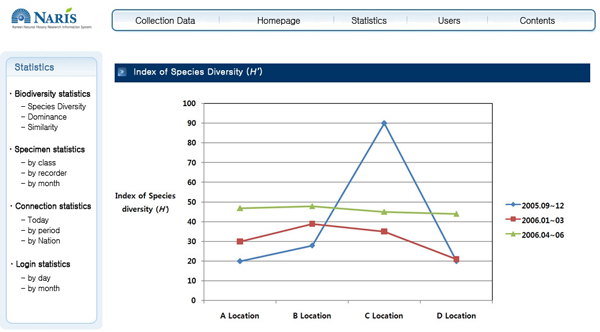
**Statistical System**. The X axis represents the location of survey, and the Y axis represents the index of species diversity. Each line (triangle, rectangle, and circle) indicates the index of species diversity for each survey period.

The data error report function has been implemented to constantly monitor and control the data quality. If a user finds incorrect data (e.g., nomenclatural, taxonomic, or spelling errors) while searching data and checking the results, then the user can report the errors using the comment box (see Figure 5). In accordance with the data error management procedure shown in Figure 6, the message is reported to a NARIS administrator, and subject area experts (e.g., taxonomic specialists) assess it and revise the incorrect data. However, the records of the modified data are kept and made visible, so qualified researchers can compare and correct them as necessary. This function is necessary for concerted efforts to improve the quality of the data. All data have errors, but that should not be a reason not to use the data, but to ensure that the error is documented and that users are made aware of the errors so that they may determine the fitness for the use of the data [25,26]. As shown in Figure 7, NARIS also has a useful function for statistical analysis that provides the reference materials for scientific research and education on biodiversity, including species diversity, dominance, and similarity.

### Future development

The future development of NARIS will contain several new functions. First, a personalized web-service (e.g., an individual database management system) will be included to attract voluntary participation of individual users. Through this system, individual users will be able to upload their own high quality and quantity of data, which will be registered on the NARIS with a verification process. Another multi-media function such as sounds will be provided for access to more exciting services. Two other new functions will be a system to distribute the actual collections and data contents, which can be actively utilized in academic researches or industrial applications, and support for taxonomic studies by providing not only morphological data but also DNA barcode data that have been recently used to identify species. Finally, molecular and genetic information that links to other bioinformatics portals, such as European Bioinformatics Institute (EBI) databases, will be offered.

## Conclusion

With the advancement of information technology, the Internet, and rapid gene sequencers, managing biodiversity information in association with genetic information is becoming a global issue. Sharing and disseminating biodiversity information among different countries through relevant international organizations is becoming the standard practice.

In this paper, we presented an effective data management system, NARIS, which is a centralized data portal enabling an integrated information retrieval of the distributed biodiversity data and is linked with genetic information from NCBI. NARIS was specifically developed in Korea but is applicable to other nations. NARIS uses the DiGIR protocol with Darwin Core2.0 format as its metadata standard for data exchange to integrate and share the biodiversity data among organizations in Korea and abroad and to play an important role as a data node of the GBIF as well.

Considering the growing importance of biodiversity resources as the original source of bio-industry, establishing this integrated biodiversity information system is expected to be integral in protecting valuable national natural resources. Also, the system will be useful for the study of species and their distributions, conservation, scientific research, education, natural resource management, climate change, social and political uses, and medicinal studies.

## Competing interests

The authors declare that they have no competing interests.

## Authors' contributions

WKP directed the study and helped draft the manuscript. JB, HMO, CBK, and YHP were involved in reviewing and critically revising it for intellectual content. JHL conceived the study and wrote the manuscript. All authors read and approved the final manuscript.
